# A ‘Textbook Pattern’? Malaria Control and Eradication in Jamaica, 1910–65

**DOI:** 10.1017/mdh.2013.20

**Published:** 2013-07

**Authors:** Margaret Jones

**Affiliations:** Research Fellow and Deputy Director, Centre for Global Health Histories, Department of History, University of York, Heslington, York YO10 5DD, UK

**Keywords:** Jamaica, Malaria, Control, Eradication, World Health Organisation

## Abstract

In 1965 Jamaica was declared free of malaria by the World Health Organisation (WHO), thus ending centuries of death and suffering from the disease. This declaration followed the successful completion of the WHO’s Malaria Eradication Programme (MEP) on the island, initiated in 1958. This account first explores the antecedent control measures adopted by the government up to the MEP. These, as advocated by the previous malaria ‘experts’ who had reported on the disease on the island concentrated on controlling the vector and the administration of quinine for individual protection. Although Jamaica suffered no catastrophic epidemics of island-wide scope, malaria was a constant cause of mortality and morbidity. Major change came in the wake of the Second World War within the changing political context of national independence and international development. In 1957 the Jamaican government joined the global WHO programme to eradicate malaria. The Jamaican campaign exposes many of the problems noted in other studies of such top–down initiatives in their lack of attention to the particular circumstances of each case. Despite being described as ‘a textbook pattern’ of malaria eradication, the MEP in Jamaica suffered from a lack of sufficient preparation and field knowledge. This is most obviously illustrated by the fact that all literature on the programme sent to Jamaica in the first two years was in Spanish. That the MEP exploited the technological opportunity provided by dichlorodiphenyltrichloroethane (DDT) with advantage in Jamaica is not disputed but as this analysis illustrates this success was by no means guaranteed.

## Introduction

At the end of 2006 Malaria in Jamaica hit national and international media headlines for the first time in over forty years when twenty-one people in the island were confirmed as suffering from the disease. From November 2006 until June 2007 a total of 368 cases were reported. In response the Ministry of Health embarked on an ‘intense epidemiological and entomological thrust’ to break the chain of transmission. The full panoply of anti-malarial measures came into operation: ‘early case finding, prompt treatment, continuous vector control, public education, personal and individual protection, and inter-sectoral collaboration and partnerships at the national, regional, and international levels’.^1^ The last of these included a grant of $6 million from the United States (US) Agency for International Development (USAID) towards the cost of equipment for spraying.^2^ Given the world’s annual death toll from malaria such case numbers appear almost insignificant and there were no deaths; however, in 1965 the WHO had declared Jamaica free of malaria and in the intervening years, apart from the odd imported case, the island had remained free of the disease. The spectre of its return thus explains this response especially given that in 2006 its return threatened the island’s biggest money earner: tourism.

Malaria is one of the world’s major killers. James Webb has argued that ‘an estimated 300 and 500 million people suffer bouts of the disease each year’ and that there are between ‘1.1 and 2.7 million’ deaths per year, and as Randall Packard has noted one child in Africa dies every thirty seconds.^3^ It had been a global disease but as it lost its hold on the temperate world, it was redefined as the paradigmatic ‘tropical’ disease and became a disease of poor underdeveloped countries. The discovery of the exact causes of malaria in the1880s and 1890s by Alphonse Laveran, Ronald Ross and Giovanni Battista Grassi^4^ opened up the possibility of more effective methods of controlling the disease and generated a debate about the optimum means of doing so which has still not been settled to this day. Malariologists from the beginning of the twentieth century adopted two broad approaches. The first was to attack the parasite in the human host through drugs such as quinine; the second was to attack the vector – the mosquito – through the destruction of its breeding environment and limiting its access to the human host through screening and nets. The former strategy, as Packard points out, was cheaper but required the cooperation of the population and, since the side effects of quinine, for example, could be very unpleasant, compliance once the immediate symptoms had subsided was not guaranteed. Destroying the vector often entailed expensive engineering works and so could be very costly and hence governments, central and local were reluctant to fund such schemes.^5^

There was a possible third approach. As Packard has argued, both these two approaches were ‘rooted in a biological construct of disease that largely neglected the underlying social and economic conditions, which …often drove the transmission of malaria’.^6^ Reducing social and economic inequalities with a view to eliminating poverty requires a fundamental re-think of the structures of any given society but even so this approach was never completely absent from the malaria debate. Webb and Packard in their recent global histories of malaria have shown how all these approaches interplayed with each other over time. One approach never entirely eclipsed another but at different times and in different places one paradigm came to predominate. These approaches focused on the control of malaria. However, in a relatively brief interval in the mid-twentieth century and for a variety of technological, economic and political reasons health policymakers considered the global eradication of malaria as a feasible and achievable objective.

In 1954 the XIV Pan American Sanitary Conference (PASC) accepted Resolution XLII which committed it to the eradication of malaria from the Americas. The newly created World Health Organisation (WHO) had established an Expert Committee on Malaria in 1947 to assist governments in setting up effective malaria control programmes,^7^ but then in the year following the PASC resolution the Eighth World Health Assembly was persuaded that the World Health Organisation should go even further and ‘take the initiative to provide technical advice and encourage research and coordination of resources in the implementation of a programme whose ultimate objective was worldwide eradication of malaria’.^8^ In 1950 United Nations Children’s Fund (UNICEF) too had shifted emphasis from providing mainly for the emergency needs of children (mainly in war devastated Europe) to long-range care programmes particularly in underdeveloped countries. These included assistance for maternity and child welfare work as might be expected but also for ‘insecticides, penicillin, vaccines, transport and sprayers for control of communicable diseases largely affecting children’ of which malaria was undoubtedly a major one.^9^ These three organisations drew directly (often in personnel^10^ ) on the experiences and methods of the American philanthropic International Health Division (IHD) of the Rockefeller Foundation (RF) active in health campaigns from the early 1900s and as it turned out they also received the greater part of their funding from the US government.^11^

The switch from the hitherto limited objective of control to eradication grew directly out of the discovery of what appeared to be greatly more efficacious insecticides against the malaria vector. In the closing years of the Second World War the apparently successful use of dichlorodiphenyltrichloroethane (DDT) in Italy to finally rid the country of malaria encouraged the scientific and medical community to envisage the possibility of conquering this major public health problem and obstacle to socio-economic development.^12^ Early successes in some parts of the United States of America (USA), Greece, British Guiana and Ceylon encouraged a sense of optimism that at last science and technology could win this war against nature’s scourge. However, as Frank Snowden has argued, the success in Italy was due to a variety of factors of which DDT was just one. These factors included improvements in housing, sanitation, diet, mass quinine distribution, universal literacy, and an infrastructure of health care services.^13^ These factors were largely ignored in the subsequent eradication campaign with its emphasis on the insecticide. The first indications of resistance of the mosquito to DDT in the 1950s instead of inducing a note of caution merely gave a vital urgency to this new aggressive policy of eradication rather than control. As Gordon Harrison argued, ‘the essential idea was to overwhelm the enemy before she had time to breed out invulnerable generations’.^14^

This optimism was of course misplaced and these efforts have been heavily criticised from a variety of perspectives. The indiscriminate reliance on insecticides – in particular DDT – proved to be extremely damaging to the environment and arguably directed expert attention and effort away from the disease itself.^15^ It also underplayed the significance of socio-economic factors in the incidence of the disease. It imposed an inflexible top–down approach, hence as Najera highlighted ‘[t]he operational aspects of the programmes were emphasized while the ability to identify and solve problems was often neglected’.^16^ Furthermore, Warwick Anderson has argued, such interventions should be seen as part of the colonial legacy, in that the ‘colonial civilising process’ was subsumed within ‘development discourse’.^17^ In the context of the cold war too this apparently humanitarian impulse can also be considered as playing its part in the wider geo-political struggle; this was particularly pertinent when it came to the Caribbean and the Americas.^18^

## The Political, Social and Economic Context

Jamaica was a British colony from 1655 until 1962, hence its economy, political and legal institutions and culture had been formed by that association. This combines with that other major determinant of the Jamaican experience: the slave system. Slavery was abolished in 1834 and full emancipation achieved in 1838 but arguably Caribbean societies in the post-emancipation era were largely shaped by the legacy of the slave system and its twin, the plantation mode of production. The ending of slavery did not inaugurate a period of social, political or economic transformation. The white elites ‘continued to monopolize ownership of the major economic resources, to exercise political ascendancy (subject to the colonial powers) and to enjoy the greatest social prestige’.^19^ The ending of slavery thus changed the political and economic context only marginally at first; and subsequently only gradually. As Hilary Beckles has argued, ‘Emancipation’ was a ‘process, rather than an event’.^20^ In addition, with the slow decline of Britain as an imperial power in the twentieth century, Jamaica along with the rest of the Caribbean came under the increasing influence of the USA. The geopolitical position of the island in the Caribbean thus ensured that its experience was invariably determined by more powerful nations.

Fundamental change in Jamaica’s political position came out of the upheavals of the 1930s and the Second World War. In the second half of the 1930s the British West Indian colonies experienced a wave of social and economic unrest which spurred the imperial government into setting up the Moyne Commission to look into their social and economic conditions. The 1940 Colonial Development and Welfare Act, which resulted from the Moyne recommendations, represented a reversal in Colonial Office (CO) policy: colonies were now to be assisted by the imperial government through the Colonial and Development Welfare Fund (CDWF) to have the services they needed, not just those they could afford. In 1944 Jamaicans gained universal suffrage to an elected Legislative Assembly and embarked on the road to independence, achieved finally in 1962. These post-war changes created a very different context for the health care services in that Jamaicans themselves were increasingly responsible for the health of their population; the policymakers were now accountable to their electorates.^21^

Jamaica’s unifying motto adopted on independence ‘Out of Many, One People’ acknowledged the diverse origins of its population and expressed a hope for future unity. The original inhabitants of the island, the Tainos, were wiped out within a few decades of Spanish occupation in the sixteenth century. The exploitation of the island for sugar cultivation by the British resulted in the enslavement and transplantation of African peoples who were by far the majority population group.^22^ Jamaican society was thus hierarchical where race and class coalesced. Whites at the top, a middle class of light-skinned Jamaicans and the majority dark-skinned working class. Jamaica was a fertile island and its soil and climate proved particularly suited for cultivating sugar, bananas, and coffee. It was a producer of primary products and thus subject to the vagaries of the world economic system. Hence from the end of imperial protection for sugar in 1846, despite its natural advantages, Jamaica suffered from a depressed economy. Such wealth as existed was concentrated in a few hands. Most Jamaicans lived in rural poverty, combining subsistence agriculture and casual labour on a diet often sufficient in calories but lacking in protein and vitamins. Malnutrition up to the end of the period was a consistent contributor to ill health. It was hence a migrant population in the search of the means to survive: from the country to the city; from Jamaica to Cuba; the mainland countries of central America and the US. Jamaican workers were subject not only to economic pressures within their own island but also to those of the world economy.

Jamaica’s major enduring legacy was that of slavery and its social and cultural impact. This undoubtedly affected in a variety of ways the development of Jamaica’s health-care services. The unwillingness of the colony’s European elite to finance such services for the majority Afro-Caribbean population was only too apparent. For example, Table 1 illustrates that, even in comparison with other West Indian colonies, expenditure on the medical services in Jamaica was still at a low level by the late 1930s.^23^

**Table 1:** Comparative salaries, size of medical establishments and ratio of Medical Officers (MOs) to population in 1938 for Jamaica, Trinidad and British Guiana. Source: NA: CO 950/47, Dr Arthur O’ Brien, *Memorandum on the Medical Services*, 3, 4, 6–7.

	*Highest MO salary* £	*Average MO salary* £	*Size of medical establishment*	*Ratio of MOs to population*
Jamaica	800	400	78	1:14,600
Trinidad	864	570	39	1:11,500
British Guiana	1056	672	36	1:8,000

Unlike many other colonial medical services, the Jamaican medical service was part-time. There was no medical education available anywhere in the British West Indies which had to be obtained expensively in the United Kingdom (UK), Canada or the US. Salaries were inadequate (private practice was both expected and necessary) and conditions of service poor. It thus proved difficult to recruit from the UK and Jamaican-born doctors were employed in some numbers but they were denied access to the top posts.^24^ They were Jamaican but of the social elite – prominent in asserting national autonomy but nevertheless limited by their class attitudes towards the majority population. Too often in the sources, too, medical officers, local and colonial government officials and the social elites shifted responsibility for disease and hardship onto the sufferer. The majority Afro-Caribbean population had to contend with perceptions created by their class, race and their slave history.

In 1920 Jamaica still had the classic health profile of a poor undeveloped country. Life expectancy was 35.9, parasitic and infectious diseases accounted for seventy-one per cent of total deaths and the infant mortality rate was c174 per 1,000 live births.^25^ The major preventable diseases were malaria, hookworm, yaws and tuberculosis. Hookworm and yaws were seldom fatal but they contributed to the low resistance of the population to other diseases. A leading cause of death was tuberculosis and in the pre-antibiotic era the only ‘cure’ for this was good food and rest – not a feasible option to most of those who succumbed to it. Malaria was both a cause of morbidity and mortality and again increased the population’s susceptibility to ill health. Economically depressed, a poor and unhealthy rural population, neglected by its imperial masters and in America’s backyard, Jamaica presented the ideal ground for the interventions of international health agencies, the first of these the IHD of the RF was involved in various vertical health campaigns on the island from 1919 to 1951, beginning with its ‘entering wedge’ – a hookworm campaign.^26^ With a population of about 600,000 in 1881 rising to c1.3 million by the end of the period, Jamaica was the largest of the British West Indian colonies; as such it serves as an exemplar of both the British colonial Caribbean experience and of the initiatives of international agencies as it moved from colony to nation.

## Malaria Incidence

As Tables 2 and 3 illustrate, malaria was one of the most significant health threats to the population in Jamaica in the colonial period. There were no island-wide epidemics on the scale of, for example, the Ceylon epidemic of 1934–5 when an estimated 100,000 people died, but it took a steady toll of life and was certainly a major cause of morbidity. It was thus a disease of attrition with no major epidemic events to motivate proactive government action.^27^

**Table 2:** Death rates for malaria and other fevers for selected years, 1878–1927. Source: Mark F. Boyd and F.W. Aris, ‘A Malaria Survey of the Island of Jamaica, B.W.I.’, *American Journal of Tropical Medicine*, 1–9, 5 (1929), 309–99, compiled from Table II, 333–4.

*Year*	*Deaths: malaria and malarial fever*	*Death rate per 100,000*	*Deaths: ague, intermittent and remittent fevers*	*Death rate per 100,000*	*Deaths: undistinguished fevers*	*Death rate per 10,000*
1878–9	—	—	139	25.0	1,056	18.9
1882–3	—	—	120	20.2	1,857	31.4
1887–8	108	17.4	214	34.4	2,004	32.2
1891–2	141	21.6	199	30.4	2,499	38.2
1895–6	170	24.6	213	30.8	2,489	35.9
1899–1900	211	29.0	197	27.0	2,960	32.8
1903–4	444	57.8	—	—	3,353	43.6
1907–8	635	78.8	—	—	4,814	59.8
1911–12	490	58.7	—	—	2,715	32.4
1915–16	483	57.2	—	—	3,486	41.2
1919	392	46.0	—	—	3,321	38.9
1923	356	41.2	—	—	3,372	39.1
1925	476	53.8	—	—	3,559	41.3
1927	358	41.1	—	—	3,218	36.7

**Table 3:** Malaria data 1927–35. Source: Taken from Jamaica National Archives, IB/5/77/173 Malaria – Marked Increase, 1936, SMO to Colonial Secretary, 21 September 1936.

*Year*	*Total cases treated as in-patients in all hospitals*	*Total cases treated as out-patients at all hospital excluding Kingston public hospital*	*Total deaths in all hospitals*	*Total deaths on the island*
1927	1,529	2,483	49	358
1928	1,587	1,888	58	330
1929	1,618	2,278	68	343
1930	1,475	2,957	42	305
1931	2,661	4,623	119	534
1932	2,470	5,496	109	423
1933	4,963	10,083	157	513
1934	4,970	14,363	156	680
1935	3,483	13,185	141	571

## Prout’s ‘Sanitary Paradise’

Malaria first received the attention of the colonial and imperial governments with the survey by the colonial expert Dr W.T. Prout in 1909.^28^ He concluded that the principal breeding places for malaria on the island were: ‘swamps and pools, shallow ditches and gutters, ponds caused by surface drainage, accidental and temporary pools, wells, drainage trenches and irrigation canals’.^29^ The malarial mosquito, thus, was concentrated on the lowland coastal areas. He estimated that, for the period from 1898 to 1907, deaths from malaria accounted for 19.7 per cent of all deaths and given that malaria was practically non-existent in the higher parishes then those deaths were concentrated in certain localities.^30^ For example, the mountainous parish of Manchester had the lowest death rate – 1.6 per 1,000; and St Thomas, where the rivers Yallahs, Negro, Morant and the Garden flowed into the sea, the highest at 6.5 per 1,000. He also calculated that one-third of admissions to hospital were due to malaria and that this estimate did not take into account those admitted for other causes complicated by malaria.^31^

Prout pointed out that malaria deaths were not the only problem; morbidity as a result of the disease also had its effect on the ‘general health and physique of a community’ and it interfered with ‘efficiency for industrial purposes’ and was a ‘considerable financial strain on the colony’.^32^ Furthermore, the figures he had collected were an underestimate of its incidence. ‘A great many’, he argued, ‘suffer from malaria and recover, and a large proportion of these receive no hospital treatment’. To uncover these hidden numbers he conducted a small spleen sample of school children and found one quarter of those he examined had enlarged spleens.^33^

He recommended, first, the drainage of mosquito breeding sites near areas of habitation, the clearing of riversides, irrigation canals, drainage canals, ponds and the oiling of water surfaces. Secondly, legislation was needed to make it a punishable offence to have mosquito larvae in private compounds. Third, he proposed measures of individual protection such as mosquito nets (expensive and beyond the means of ordinary Jamaicans) and the screening of buildings. He noted there was only one mosquito-proofed building on the Island: the Police House at Port Henderson.^34^ He also drew attention to the dramatic decline in malaria in Italy, which many contemporaries attributed to its state quinine policy^35^ and argued that quinine should be systematically administered to certain specific groups accessible to government contact – the police, indentured labour and schoolchildren.^36^ As far as the general population was concerned they could not be forced to take quinine but they could be encouraged to do so by having it freely provided and easily available. As to the cost of these measures, he argued that it would be recouped within three years by savings in hospital expenditure alone.^37^ If these recommendations were followed, he argued, then Jamaica could indeed become a ‘sanitary paradise’.^38^

As a result of his survey a Malaria Commission was appointed in October 1909 to ‘investigate and take measures to remedy the conditions that give rise to Malarial Fever on the Island’.^39^ However, only palliative measures within the means of administrative bodies or individuals were undertaken.^40^ The screening of public buildings was limited; seventeen police stations had been screened but the hospitals at Buff Bay, Hordley and Montego Bay were the only ones where screening (and only partial screening at that) had been done.^41^ Prout’s recommendations on the distribution of quinine were adopted but similarly with little real commitment by the government. Systematic doses of quinine were given to accessible, necessary and economically significant groups of people such as the police and the labourers on the estates where employers were compliant. In the latter case at least it seems that labourers found their own way to oppose this compulsory medication. More than one overseer said it was administered but that the labourers refused to swallow it. For example, in answer to the MOs’ questioning one overseer complained that it was ‘given regularly but you will find that it will all be in the yard as the coolies pretend to swallow it and go into the neighbouring yard and spit it out’.^42^ Some free quinine was also distributed through the schools but that depended on the willingness of the teachers. As the Inspector of Schools of St Mary’s reported, although it had been ‘very beneficial to the children’s health’ and that the ‘increased school attendance …was entirely due to it’; in many instances ‘teachers took little or no interest in the matter and the quinine remained undistributed’.^43^

As far as the general population was concerned the passing of two laws in early 1910 – a Tariff Law and a Sale of Drugs and Poisons Law (both amendments to previous legislation) – reduced the cost of quinine and permitted its sale by any unlicensed person. This facilitated the sale of quinine through Post Offices at a very cheap rate but it was not free as Prout had wanted. In fact the price allowed some profit as well as a bonus of ten per cent to the Post Mistress. As the Superintending Medical Officer (SMO) accepted in his 1910 report: ‘it remains to be seen whether much will be sold, and whether the people will take advantage of the opportunities to buy cheap quinine’.^44^ In the absence of symptoms, quinine’s unpleasant side effects understandably militated against its general take up. This first Malaria Commission was short-lived. It was disbanded in 1913 most probably due to lack of funds.^45^

## The International Health Division and Malaria Control

The second major malaria initiative on the island was undertaken under the auspices of the IHD of the RF. A rise in malaria deaths in the mid-twenties precipitated once more an interest in malaria control. In September 1927 the Legislative Council voted the sum of £1,200 towards their share of the cost of a malaria survey^46^ and on 9 January 1928 Dr Mark F. Boyd, Director of Malarial Studies at the RF, arrived in Kingston to begin his work.^47^ Dr Boyd’s report, co-authored with his Jamaican deputy Dr F.W. Aris cover much the same ground as Prout’s survey twenty years previously.

Boyd and Aris concluded that endemic malaria ‘is largely, if not altogether, confined to the coastal plain lowlands’ which, as they pointed out, comprised some of the most valuable economic areas of the island: the sugar and banana growing areas.^48^ In these areas malaria transmission was frequent and common but did not manifest any striking changes in intensity from year to year. It was in the larger zones of lesser intensity where striking differences in intensity appeared. Lower rainfall in these areas produced a noticeable drop in cases but it was these areas where epidemics were likely to occur. There was little if any malaria transmission over the interior or the greater part of the island principally because of the distribution of the vector. However, smallholders from the inland areas annually migrated to work on the lowland estates for part of the year. They were particularly susceptible to the disease and also carried it back with them.^49^

Boyd and Aris’s extensive field work identified the malaria vector and its distribution throughout the island and found that the vector *A. albimanus* gave the greatest cause for concern:^50^

Its distribution was widespread over the lowlands…. It will avail itself of temporary water …. The period of its annual maximum prevalence is during the summer and fall, coinciding with the principal periods of malaria transmission…. It is a species which satisfies its hunger on human blood [and] it has proven itself susceptible to infection with the parasites of malaria.^51^

The most common type of parasite found was quartan malaria (*P. malariae)* which was in line with investigations conducted elsewhere which showed that a reduction in the intensity of malaria led to its increase in relation to the other parasites of *P. falciparium* and *P. vivax.* ^52^ They questioned the effectiveness of the government’s quinine policy. An analysis of Post Office sales made by the Superintending Sanitary Medical Officer (SSMO),  Dr Strathairn, in 1922–3 had shown, first, that the volume of sales bore little relationship to the distribution of malaria. The highest volume of quinine sales were in towns which were important market centres but not necessarily closest to endemic foci. Second, a comparison of annual total quinine sales showed no fluctuations that paralleled the incidence of malaria. Lastly, the actual volume of quinine distributed through the Post Offices considered ‘from the standpoint of its consumption by malaria patients, rather than per individual of the population, is too small to expect that many malarious persons have actually taken the remedy in effective quantities’. It was therefore their opinion that

the volume of quinine consumption since 1910 has been too small to warrant a conclusion that there has been any relationship between the inauguration of this policy and the present declining trend in malaria incidence, which had its beginning at about the same time.^53^

Hence they argued that, although the government should continue the policy of Post Office sales, the ‘practice as carried out in the past, or as may be continued in the future, should not be regarded as an effective means of dealing with the malaria problem of the island’.^54^

Their recommendations continued the focus on control of the vector. Control could be maintained through dusting with Paris green, reclamation and drainage works. In addition, malaria should also be made a notifiable disease; increased accuracy of diagnosis should be achieved through a public health laboratory which would raise the standards of treatment and provide better information on malaria incidence; and there should be more public education on malaria, its transmission, and how to prevent it.^55^ There was some reference to the role that housing played in malaria transmission but it was seen as a technical not a social problem. They recommended the duty free importation of wire screen cloth to encourage mosquito proofing of houses and the lowering of tax assessments for those householders who complied with sanitary requirements.^56^ More drastic measures were recommended for rural inhabitations and estate housing. Italy may again have been the model here as Boyd and Aris advocated ‘the relocation and concentration of dwellings and barracks on estates’ as far away as possible from anopheline breeding areas, and then ‘giving attention to the proper orientation of their doors of their doors and windows to the prevailing winds, and the study of methods of economical mosquito proof construction’.^57^ The wishes of these rural householders were not considered an issue. ‘The head men of the villages should be obliged to enforce the cooperation of the occupants’.^58^

Like Prout’s before it, the Boyd/Aris malaria survey prompted the government to implement anti-malaria work. Early in 1929 the Legislative Council (LC) voted £4,000 for the work and a Malaria Commission was established.^59^ The RF made a small financial contribution of about £200 and also provided a replacement for Dr Aris, Dr Paul Carley, while the former was on a RF scholarship in Puerto Rico. The commission’s remit was to conduct further research, and to put ‘into field application those measures which prove to be of practical benefit in the control of malaria’: initially this would be in certain limited areas so that lessons learnt from that could then be applied elsewhere. By 1930 there was still much optimism about the success of the Malaria control programme. Paul Carley reported that over 800,000 square yards was dusted with Paris green during the first half of 1930, two miles of tile drainage put in, and surface drainage carried out over a 100 miles of ditches. One thousand cases had been treated with Plasmochin and quinine and 1,400 blood examinations done.

Unfortunately, the projected success of this control programme hit a natural obstacle. After several years of low rainfall, which Boyd had argued accounted for the declining incidence of the disease, the 1930s was a period of unusually heavy rainfall and the incidence of malaria increased again, so much so that in Falmouth on the north coast of the island it was termed an epidemic. Despite these control programmes, in 1937 Dr H.M. Johnston who took over as Director of the malaria campaign in that year concluded that ‘With the present limited programme of control, malaria promises to remain one of the foremost public health problems of the Colony for some time to come’. What was needed, he argued, were more permanent measures of drainage and filling, instead of the temporary measures which were so often resorted to.^60^ This narrow focus on the technology of malaria control contrasts with the case of Ceylon where the 1934–5 epidemic had led to an island-wide Malaria Control and Health Scheme in 1937. This, as well as these usual direct methods, included measures designed to deal with the ‘conditions which aggravated the incidence of malaria’ such as maternal and child welfare work, health education and general sanitary work.^61^ In Jamaica, however, malaria was therefore one health threat amongst many others to be dealt with by a medical system which paid little attention to preventive health services.^62^

The political context for malaria control policies in Jamaica changed fundamentally after 1940. The 1940 Colonial Development and Welfare Act abandoned the principle of colonial self-sufficiency and established a fund which allowed for the imperial financing of welfare projects in the colonies. The British West Indies (BWI) were the first recipients of this imperial largesse and in 1943 under scheme-D113 of the CDWF, Jamaica was given £38,235 over a period of five years (extended for another five years in 1948) for malaria control work. This funded a permanent Insect Control Service responsible for malaria control in all its phases including residual sprayings, drainage and larviciding and a Malaria Research Laboratory to examine all blood smears collected from surveys and to conduct entomological research.^63^

By 1946 there were twenty-three control areas and a limited house spraying had been attempted. Although this gave ‘encouraging results’, the supply of DDT was ‘too inadequate and spasmodic to provide any useful statistics’.^64^ This residual house spraying continued: in 1949, for example, 1,207 houses were sprayed but there were still no definite conclusions as to its effectiveness.^65^ In 1950 the CO sent two experts to the island, Dr Muirhead-Thomson to study the malaria problem followed by C.B. Symes, from the Colonial Insecticides, Fungicides and Pesticides Committee. Symes recommended that, since there had been no malaria survey since Boyd and Aris in 1928, then that lack should be rectified but, second and more significantly, he advocated seeking ‘financial and technical assistance from organisations outside the island which could command larger resources’.^66^ The resulting 1950–1 malaria survey consisted of spleen and parasite examinations in 338 schools, a house-to-house fever and blood smear survey and an investigation into the prevalence of the mosquito within a mile radius of the schools. By July 1951 137 schools had been surveyed. The spleen index in schools was 23.4 per cent with 26.5 per cent positive blood smears in the ‘controlled areas’; 14.5 per cent and 23 per cent in ‘uncontrolled areas’.^67^ It had proved impossible to complete the analysis of the data from the house-to-house survey as ‘difficulties in the way of laboratory facilities were insurmountable’ so only fifty-nine per cent of the smears taken had been processed, of these 670 were positive.^68^ However, the adoption of Symes’ second recommendations did indeed prove to be a turning point. In 1952 the Jamaican Government, UNICEF and Pan American Health Organisation (PAHO)/WHO agreed to carry out an island-wide insect control programme based on DDT house spraying which was designed to eradicate *aedes aegypti* and control malaria.^69^ The government provided the legislative framework in the following year; malaria was finally made a notifiable disease and regulations were laid down for the spraying of private houses. Spraying could take place at forty-eight hours notice between the hours of 7am and 6pm. Householders were expected to remove or stow all items which could be damaged, extinguish all fires and leave all receptacles intended for the storage of water accessible for inspection. If the occupier of the premises could not be found or was unknown within seventy-two hours then forcible entry was permitted.^70^ There is no available evidence on the extent to which this last stipulation was evoked.

The success of these control measures as evidenced in Table 4  encouraged the WHO/PAHO and the Jamaican government to move from malaria control to the eradication of the disease as part of the global MEP. Jamaica was to participate in the campaign to finally rid the Americas of malaria.

**Table 4:** Numbers of houses sprayed, positive blood smears and malaria deaths, 1952–7. Source: Compiled from WHO7.077 parasitology archives, register of malaria eradication, tables, 1, 6, 7, pages 6, 10, 11.

*Year*	*Number of houses sprayed*	*Blood smears examined*	*% Positive*	*Rate of malaria deaths per 100,000*
1952	22,736	11,189	32.54	49.7
1953	41,685	22,338	29.86	43.0
1954	171,421	20,038	14.23	35.6
1955	191,263	21,277	4.85	17.0
1956	259,030	13,916	2.85	17.7
1957	152,080	8,922	2.97	14.3

## Jamaica and the MEP

According to the *Register of Malaria Eradication of Jamaica* (the concluding report issued by the WHO/PAHO on the completion of the eradication programme) the project had gone entirely to plan – an impression reinforced by the words of the director of the island’s medical services, Dr H.M. Johnson, who highlighted that it had followed the ‘textbook pattern’ for eradication.^71^ That pattern had been laid down in the sixth report of the WHO Expert Committee on Malaria in 1956. It proposed four phases: preparation (surveys, training of personnel and a pilot study); attack (mass spraying of houses); consolidation (active case finding and treatment); and maintenance (necessary until global eradication had been achieved). It estimated that the first three phases should take between five and eight years.^72^ As described in the *Register*, Jamaica appeared to have followed the blueprint in an exemplary fashion.

The tripartite agreement between the Jamaican government, WHO/PAHO and UNICEF was signed in 1957 whereby Jamaica received technical assistance from PAHO/WHO and supplies from UNICEF and in return committed itself to maintaining the programme through detection, eliminating the foci of infection, health education, a full laboratory service and ultimately integrating the programme into the general public health services. This entailed funding the operational activities and the salaries of the personnel.^73^ The programme was based on attacking malaria transmission in the home where both man and mosquito were infected, hence it was the ‘number of houses in a malarious area’ which was the ‘important factor in planning MEP, rather than the type of mosquito breeding areas or number and severity of malaria cases’. In addition the programme was to give the same consideration to sparsely populated areas with low grade endemicity as was given to densely populated highly endemic zones as they otherwise remained a source of re-infection.^74^

The eradication programme was planned meticulously on paper. The period of preparation began toward the latter part of 1956 and continued until September 1957 when it would move into the period of full coverage; the attack phase would last four years commencing in January 1958, to be followed by the third phase of vigilance and prevention termed the maintenance phase. The target population in malarious areas was estimated to be 1,282,700 (total population 1,656,043) with 298,411 houses to be sprayed. With the assistance of the WHO the government had drawn up a map of malarious areas with the extent and means of communications and the distribution of the populations and houses. The Malaria Eradication Service was to carry out the plan as part of the Insect Control Service under the direction of the chief medical officer. It consisted of three departments: spraying operations, evaluation and administration. The island was divided into three zones for spraying, each under a zone supervisor. Spraying was carried out initially by 135 spray men organised in thirty-two teams. A public health inspector was assigned to supervise the spraying and the teams were based at the parish health office and travelled each day to their area of spraying. Evaluation consisted of twenty-six evaluation officers assigned to the parishes and a malaria research laboratory. The administrative department was responsible for personnel, warehousing, supply operations, transport and its maintenance.^75^ The Jamaican government’s financial commitment was estimated to be £477,945 of the total cost of £683,434. UNICEF pledged to provide the remaining £205,489 to cover the costs of insecticides, spraying equipment, microscopes, vehicles and protective clothing.^76^

To supplement the MEP, Kingston was also home to The Malaria Eradication Training Centre (METC) established in April 1958 in Kingston. This was the first international training centre of its kind for English-speaking students and was jointly sponsored by the International Cooperation Administration (ICA)/US Agency for International Development (USAID), PAHO/WHO and the government of Jamaica. It offered senior eleven-week courses for physicians, entomologists, engineers and other post-graduates and junior eight-week courses for public health inspectors, sanitarians and technicians. By 1964 409 students had trained there from sixty-nine different countries in the Americas, Africa, Europe Asia and the Western Pacific.^77^

The attack phase began on 2 January 1958. Nearly two years later at the end of 1959 Dr Ross Nicol, the PAHO/WHO malaria consultant, arrived in Jamaica to assess the progress of the campaign. His first report, after two years of the campaign, was highly critical. It highlighted four major problems: poor communications between the various responsible bodies resulting in a great lack of overall direction; deficiencies in the system of reporting and detection; severe transport difficulties; and the negative attitude of the population to spraying activities. Some of these problems were easier to fix than others but all in varying degrees suggest a lack of proper assessment of the particular circumstances existing in Jamaica. As a representative of the WHO who had no prior experience in Jamaica, Ross Nicol’s viewpoint was necessarily top–down. However, his criticisms must carry some weight since, as a supporter of the MEP, he had little interest in highlighting faults unnecessarily, rather the opposite; and additionally his comments at times empathise with the difficulties encountered by those having to implement the work. In addition, Ross had found it ‘utterly incredible’ that his consultant report was the first that had ever been handed to the Jamaican officials and to him this explained a ‘very great deal of what had occurred in the past’.^78^

Ross Nicol found on his arrival that the Malaria Programme headquarters (HQ) was located at the Ministry of Health but the evaluation office and the laboratory in another building some distance away; since communication between the two was done through letters this had resulted in considerable delays. He tried to encourage the use of the telephone for urgent matters.^79^ A further measure he instigated in order to offset the lack of contact was a series of coordinating meetings. These included weekly staff meetings of PAHO/WHO staff, a coordinating meeting of the Jamaica national and PAHO/WHO staff every two weeks and the METC was approached with a view to improving cooperation so that all METC weekly staff meetings were attended by the Jamaican staff.^80^ He was also horrified by the office building provided for the PAHO/WHO team. He described it as being in the ‘worst condition’ he had ‘ever seen during his service’. He considered that the acceptance of these poor conditions explained ‘why the Organisation is relegated to a minor place among the other agencies here when such conditions are accepted by the staff’. He claimed that when the government was approached to improve the building they were ‘extremely helpful’; government officials consistently gave the excuse for their neglect that ‘We were never asked’. There is no way of knowing if such requests had been ignored or if the usual experience of the Jamaicans suggested that there was little point in making such requests. In response to the outsider, however, the building was completely renovated, repainted, the offices cleaned and the gardening equipment dumped in the outside porch removed. In addition Ross Nicol ensured that, as according to the agreement, the team received their permanent secretary, a new typewriter and electric calculator.^81^

The transport crisis was termed by Ross Nicol as of the ‘TOP MOST URGENCY’. There was an inadequate supply of vehicles in the first place, which meant the existing ones were overused and broke down. In that eventuality the team had to turn to the Public Works Department (PWD) or local garages. The PWD could not cope with the demand and long delays resulted for fairly simple repairs. Local garages were ‘utterly inadequate, both from workmanship as well as spare part supply’. There was also a very high accident rate in his view because the low salaries paid to drivers meant that the majority of those recruited were of inferior quality and that any good ones left. A further factor was that the vehicles supplied were not the ones originally specified by the Jamaican government and this had resulted in a diminished carrying capacity as those that were sent were smaller. Furthermore, the number of men, number of equipment items per man, and the weight of insecticide had not been factored properly. Another example of incompetence were the small Austin cars supplied to the evaluators which were not suitable for Jamaican roads, ‘being completely useless for penetrating off paved road into areas where true evaluation must be done’. ‘Unless the transport situation is relieved forthwith’, Ross Nicol concluded, ‘the campaign faces an immediate breakdown of spraying operations in a large part of the country …and the end can only be disastrous’.^82^

By March 1960 Ross Nicol reported that action on this aspect had been rapid. UNICEF had delivered three three-ton vehicles. Locally a site for a workshop for repair of vehicles was in the process of being constructed and UNICEF had cabled for information on the electrical characteristics in order to despatch appropriate workshop tools. The government was also embarking on the selection of a top grade mechanic ‘at an adequate salary to ensure his permanency’.^83^ However, this workshop did not finally open until April 1961. In June of that year too eight new jeeps were also put into operation to further alleviate the transport problem.^84^

At another basic level of field work Ross Nicol was critical of the system for employing the spray men. He was ‘astonished’, he commented, to learn that all spray men were recruited on a casual labour basis. They turned up on a daily basis to do a day’s work and were paid the same wages every day. With ‘no assurance of security and future advancement’ they had ‘little or no esprit de corps or pride in their work’. This highlighted the need for greater supervision of their spraying activities and the planning of their itineraries.^85^ It should be noted that the wages of all workers were the responsibility of the government. Initially, the insecticide used was dieldrin, to be sprayed once a year; however, this had to be changed to DDT on a semi-annual basis as resistance was found in *A. albimnus* to the former.^86^ This changeover was not made immediately but over the course of 1959 ‘because of difficulties in obtaining additional funds for the change and in reorganizing spraying personnel’.^87^

Ross Nicol also highlighted crucial problems in the evaluation procedures. The MEP entailed using the new diagnostic method of finding parasites in blood smears rather than relying on fever counting and spleen counts.^88^ This method presupposed good laboratory facilities and trained technicians - neither, as Ross Nicol found, were present in Jamaica. The laboratory facilities were seriously deficient thus creating a bottleneck in diagnosis. Only 350 slides were being processed each day although on paper there were supposed to be twelve microscopists completing fifty slides each per day. There had apparently been great difficulty in recruiting and keeping suitable personnel as young people soon lost interest and left.^89^ Reporting of cases in the field was also hampered by the system of handwritten ledgers in the parishes resulting in overwork and more delays. Ross Nicol hoped to get agreement to establish a card index type of recording although he anticipated that this would take ‘considerable time’.^90^ Again according to his second report things had improved: ‘[w]e have broken the laboratory bottleneck and the evaluation scheme can now proceed’.^91^ A revised scheme was being set up which included the establishment of a collection and replacement service for slides to ensure minimum delay in deliveries to the laboratory and the provision of adequate clerical personnel and printing of forms for laboratory use.^92^ However, although the bottleneck had been eliminated it would need, he argued, ‘careful supervision in ensuing months to ensure that capacity and quality do not deteriorate’.^93^

The other aspect of evaluation was the detecting and treatment of cases essential to stop the cycle of transmission. This became an even more important aspect of the programme once the maintenance phase was entered. When spraying had ceased some spray teams remained in each parish to continue the case detecting through house visiting and blood sampling. Local health and medical units also cooperated in taking blood smears from suspected cases. According to the *Register* ‘on average about 16,800 blood smears have been submitted annually by approximately 670 information posts on the island’ and it concluded that the ‘networks for epidemiological evaluation and malaria surveillance have been very adequate’.^94^

However, reports from the officers in the field suggest that implementing the evaluation procedures was somewhat more difficult than this. The WHO entomologist, Dr Van Seventer, who was advising on the evaluation system in March 1961, commented that the ‘weak point’ of the system was not in the spraying but ‘in the treating and finding of cases’.^95^ Ross Nicol initiated improvements in the evaluation system by arranging for the production of a short instruction manual for individual evaluators and, more vitally, establishing three-day training sessions.^96^ These sessions were held in the field and attended by senior staff. They were described as necessary for the ‘indoctrination of the evaluators’ so that they were ‘fully conversant with every detail of the task’ and that ‘no evaluator would be permitted to begin work until all members of the senior staff are satisfied as to his capabilities’.^97^ As the attack phase advanced so did the extent of the evaluation service. By April 1961 there were thirty-two evaluators and 694 reporting posts; these consisted of health centres, hospitals, government dispensaries, public health inspectors, midwives and schools.^98^

Routine spraying of the island ceased on 31 December 1961 so that from 1 January 1962 Jamaica entered the phase of ‘Consolidation Surveillance activities’.^99^ This meant the scheme of active case finding had to be maintained and improved. However, equally if not more important at this stage, Ross Nicol emphasised, was the ‘*Stimulation* and *Expansion* of *Passive* case Detection system’ which ‘up to the present, as in so many other programmes, has been difficult to achieve’.^100^ The by then forty-eight evaluators (distributed among the parishes according to size and population and composed of public health inspectors, malaria headmen and clerks) were still responsible for house-to-house, clinic, dispensary, hospital outpatients visits to find fever cases and take slides. School surveys were being reduced in favour of the former. They also, noted Ross Nicol, did a great deal of work in ‘stimulating *Informers* who, here in Jamaica, *Do Not Take Slides,* but only *inform* the Parish Headquarters by post, of any fever sufferers in the area, on specially printed cards’.^101^ He commented that approaches had been made to nurses at clinics to take slides in the hope of reducing the time spent on this task by the evaluators but ‘the results overall are not encouraging. One dislikes to substitute an order for voluntary aid but this may need to come’.^102^ This certainly suggests that cooperation from Jamaican medical professionals was not always forthcoming.

An ‘active informer service’ was vital and those currently on the list who were unproductive should be replaced, he advocated, by ‘new and more active volunteers’. Hence the current list of ‘*Collaborators’* should be revised. More detail should be given upon ‘how Collaborators are selected, how they are given the title of Collaborators, by whom they were selected, and to what extent their collaboration is of value’.^103^ He considered that ‘a clear definition of “Collaborators” as understood here in Jamaica would contribute a great deal to clarifying the present reporting, and bring it into understandable focus with other country programmes’.^104^ He cited two examples of medical professionals who were automatically termed collaborators but who did not necessarily merit their inclusion on the list. Many general practitioners took no slides at all nor had any intention of doing so and should be removed. Another group he deemed unproductive were midwives: ‘from personal experience in field interviews’ he felt they were ‘not really interested’. These ‘ladies have a large potential but appear to lack the desire to develop it’.^105^ Given that the medical services were overstretched, this task would have been one more burden. In addition, the emphasis of Jamaica’s medical services had traditionally been on the curative aspects of health care; preventive health care services had always come a poor second.^106^ It is difficult to comment confidently on the significance of nomenclature but if the MEP was to be seen as a weapon in the cold war then the terms ‘informers’ and ‘collaborators’ to describe its facilitators seems singularly ill-advised.

The most glaring failure of communication, tact and basic field knowledge originated at the very top of the MEP organisation. No one had noted that Jamaica was an English-speaking country so all key literature sent from WHO/PAHO from the start of the programme had been in Spanish. This had meant, Ross Nicol pointed out, that the national staff had ‘wasted endless hours’ working on documents not in their own language. Apart from being an unnecessary burden, ‘a very natural feeling of resentment towards the Organisation has arisen and while the Jamaicans are too courteous to make violent protests yet the fact remains that they do not like it’. Furthermore, it meant that for two years the opportunity to disseminate vital PAHO/WHO literature on the campaign to the physicians of the island was lost. Ross Nicol argued that the difficulty ‘in securing what the organisation may consider more ideal cooperation may be traced to the very natural reaction of a group of intelligent high level professional men, who are extremely busy, being supplied with what is obviously up to date information in a language they cannot read’.^107^

Given this catalogue of inefficiencies perhaps it was not so surprising that Ross Nicol and others commented on the lack of cooperation if not outright hostility the ME team received from the population at all levels. To help overcome the understandable indifference of the medical profession, Ross Nicol proposed to get together a small unofficial group of professional men such as entomologists, agricultural experts and retired professors who were interested in the campaign to provide an ‘authoritative pool of collaborators’ who could help in solving problems peculiar to Jamaica. Similarly, he hoped to interest the ‘leading ladies of the community’ to take over clerical work in the labs and hospitals thus freeing technicians for the work of microscopy. Thus initiative would, he argued, ‘go a long way to combating the unfavourable reaction shown by the public towards the spraying operations’.

Public opposition appeared to be significant. For example, he highlighted the ‘deep-seated reluctance, amounting to downright refusal’ to accept spraying by the wettable powder that was then being used in Jamaica. Although he proposed a switch to an emulsion solution to rectify the problem he feared that ‘hostility to spraying operations has now developed into a very real menace to the success of the program’.^108^ H.M. Archibald who reported to the WHO HQ in 1959 on the MEP in Jamaica also commented on the lack of cooperation displayed by the populace. The task of the spray men, he noted was ‘peculiarly difficult as the Jamaican houses are unusually full of furniture and the people are not cooperative in removing it’; there was ‘antagonism due to the staining of some emulsion paints’ with the DDT and the fact that it did not kill bed bugs, and not least there was the ‘independence of the Jamaicans who automatically react adversely to governmental direction’.^109^

These problems were doubtless not unique to Jamaica, to the MEP or to other WHO initiatives; however, they do indicate that one should be cautious about attributing undue power and influence to the WHO or any of international organisations.^110^ The intentions of those at the PAHO/WHO HQ could not easily be translated into effective action, particularly when the desire to proceed as rapidly as possible entailed limited preparation. Furthermore, the institutional and cultural drift in Jamaican governance exerted its own pull. The WHO MEP team could not override the customary lacklustre approach to the health care services in Jamaica, or extract more financial resources beyond the customary minimum. Furthermore, Jamaicans were not accustomed to accepting the dictates of authority without a degree of suspicion: their history did not encourage a belief in a benevolent government.

## Conclusion

Despite this catalogue of deficiencies at all levels of the MEP in Jamaica, it was – and this cannot be underplayed in importance – successful. The last case of *P. falciparium*, the most common form of malaria, was recorded in June 1961 and in 1965 the WHO declared the Jamaican MEP complete.^111^ Jamaica undoubtedly benefited from a moment of optimism (even at the time considered misplaced by some) when eradication was considered feasible and when at the same time access to funds for malaria control from the US the major donor depended on adopting the eradication programme.^112^ However, as is clear, the Jamaica MEP also suffered from the weaknesses exposed in other MEP areas. For example, as Cueto has argued, malaria eradication resembled a ‘belief system’: it ‘was based more on the conviction that a transnational network of brilliant people and bilateral and multilateral agencies’ could effect success than ‘empirical experience’.^113^ This contributed to the downplaying of cultural, political and social differences which in turn presented obstacles in gaining the necessary cooperation of those involved whether as agents of the MEP or recipients.

As has been noted the medical professionals in Jamaica were from the beginning, if not overtly antagonised, then at the least made to feel peripheral to the campaign, particularly by the language issue. This must have contributed to their lack of active cooperation highlighted by Ross Nicol, especially in the consolidation phase of the programme. It is also a significant illustration of the inadequacies of the top–down approach evident in other aspects of the programme. One can only guess, furthermore, at the frustrations felt by those responsible for delivering the programme with its catalogue of inadequate equipment and inefficient systems. That it was two years before these early problems were realised and rectified also suggests a degree of complacency at the top about the ease of the endeavour.

It was evident too that the MEP adopted a purely technological approach to the disease. As with the various control programmes which had preceded it, the WHO MEP concentrated on the technology of malaria control and eradication and bypassed the socio-economic factors which contributed to the prevalence of the disease. The poverty of the population was noted by all the outside specialists who went to Jamaica to report on the problem. Prout based his plea for free quinine on the inability of the population to afford it otherwise. Boyd and Aris similarly commented that, ‘while the island as a unit is very prosperous, the incomes and resources of most of the inhabitants are very small…the majority …are very poor’.^114^ The WHO entomologist Dr Van Seventer who visited Jamaica in 1960 concluded that the persistence of malaria in the parish of Clarendon, for example, was caused by people sitting outside in the evenings during the biting hours. The reason for this obviously risky behaviour was that their houses were so small they could only be occupied for sleeping.^115^ These experts acknowledged the poverty and its contribution to malaria but then offered solutions which were wholly technical. So beyond the usual public health education campaigns which were designed to inform and change individual behaviour^116^ there was no attempt to link malaria eradication to the socio-economic position of the majority of Jamaicans.

Despite these failings the MEP was successful. In many respects Jamaica presented the ideal circumstances for the MEP. As an island it presented a small unitary area which could be insulated against the reintroduction of the disease. By the 1950s it had an island-wide infrastructure of medical services with an extensive network of hospitals and health centres. With the foundation of the University of the West Indies in 1948 it had a medical college and teaching hospital. After centuries of neglect the imperial power had made available funds for welfare projects; and in the 1950s the island was moving towards nationhood. Under its new political leaders, the health care services were now the responsibility of an elected Minister of Health, and were susceptible to arguments about the economic benefits and the new freedom from disease the MEP offered. Instigated by the RF, Jamaicans had been subject since 1925 to prolonged public health education campaigns. A public health educated populace should have provided ready-made cooperative subjects. Furthermore, the preceding spraying campaign had provided a basis for the programme which could be utilised and intensified. Despite these positive features, it is however, tempting to accept Randall Packard’s claim that the WHO/PAHO successes in the Caribbean, as exemplified here by Jamaica, were as much a result of ‘small size, relative isolation, and ability to restrict the reintroduction of malaria from outside’.^117^

The success of the MEP campaign in Jamaica was a significant achievement for the WHO at a time when disillusionment with eradication was already taking over. The particular circumstances of Jamaica which clearly were not generalisable could not stem this process or offer counter arguments. However, and as important, even though malaria deaths were falling by 1950 (4.5 per cent of all deaths in 1950 – almost half those from tuberculosis^118^) it was of enormous benefit for the health of the Jamaican population. It followed the ‘textbook pattern’ in adhering to the prescribed pattern at the same time as it highlighted the common deficiencies of this pattern.

## Footnotes

1. Ministry of Health, Jamaica, Protocol for Prevention, Control and Management of Malaria, Division of Health Promotion and Protection, ‘Ministry On Thrust to Eliminate Malaria’, 4 December 2006, 21 June 2007, and 20 February 2008. Kingston. Available online at http://www.moh.gov.jm/ministryOnThrust.html (accessed 20 February 2008).

2. ‘USAID boosts malaria fight’. Available online at http://www.jamaica-gleaner.com/gleaner/20070111/lead/lead6/html, Thursday 11 January 2007 (accessed 20 February 2008).

3. WebbJames, Humanity’s Burden. A Global History of Malaria (Cambridge: Cambridge University Press, 2009), 2; Randall M. Packard, *The Making of a Tropical Disease. A Short History of Malaria* (Baltimore, MD: Johns Hopkins, 2007), xvi.

4. Webb, *ibid.*, 128–9.

5. For a detailed exploration of these approaches see Webb, *op. cit.* (note 3), 129–59; Packard, *op. cit.* (note 3), 111–49.

6. Packard, *op. cit.* (note 3), 126.

7. NajeraJ.A., ‘Malaria and the Work of the WHO’, Bulletin of the World Health Organisation, 67, 3 (1989), 229–243: 230.PMC24912502670294

8. *Ibid.*, 232.

9. www.unicef.org/about/history/files/exec_board_special_rpt_1953 Accessed 11 July 2012, United Nations International Children’s Emergency Fund, Special Report of the Executive Board 25 March 1953, 1, Paragraph 7b.

10. For example, Fred Soper who enjoyed a long career in the IHD before becoming Director of the PASB in 1959. Marcos Cueto, *The Value of Health. A History of the Pan American Health Organisation* (Washington: Pan American Health Organisation, 2007), 87–88.

11. For example, in 1959 the $9.5 million dollars for the MEP came mostly from the US and in 1961 the US provided $11.milliuon of the $11.6 million WHO funds. Marcos Cueto, *Cold War, Deadly Fevers: Malaria Eradication in Mexico, 1955–1975* (Baltimore: Johns Hopkins University Press, 2007), 66.

12. HarrisonGordon, Mosquitoes, Malaria and Man: A History of Hostilities Since 1880 (London: John Murray, 1978), 218–248

13. SnowdenFrank, The Conquest of Malaria. Italy, 1900–1962 (New Haven, CT, and London: Yale University Press, 2006), 219

14. Harrison, *op. cit.* (note 12), 236.

15. See for example, Cueto, *Cold War, Deadly Fevers*; Packard, *op. cit.* (note 3).

16. Najera, *op. cit.* (note 7), 233.

17. Warwick Anderson, *Colonial Pathologies: American Tropical Medicine, Race and Hygiene in the Philippines* (Durham and London: Duke University Press, 2006), 183.

18. Cueto, *op. cit.* (note 15), 49–67.

19. BreretonBridget and YelvingtonKevin A., ‘Introduction: the promise of emancipation’, in Bridget Brereton and Kevin A. Yelvington (eds), The Colonial Caribbean in Transition. Essays on Postemancipation Social and Cultural History (Kingston: University of the West Indies Press, 1999), 1–22: 6.

20. BecklesHilary, ‘Introduction’, in Hilary Beckles and Verene Shepherd (eds), Caribbean Freedom. Economy and Society from Emancipation to the Present (Kingston: Ian Randle Publishers, 1996), ix–x1: x.

21. JohnsonHoward, ‘The British Caribbean from demobilization to constitutional decolonization’, in Judith Brown and W.M. Roger Louis (eds), The Oxford History of the British Empire. The Twentieth Century (Oxford: Oxford University Press, 1999), 597–622

22. After emancipation when labour was again needed, indentured workers from India, China and Africa were imported into the island, but in relatively small numbers.

23. To reach the figures for the salaries I have used the conversion for Trinidadian and British Guiana dollar of $/article/back/fn-group/fn/p/mixed-citation/inline-formula  sterling (ie. c$5 to the £) given by Dr O’Brien. I have also rounded figures up for the salaries and for the population numbers.

24. Dr J.H. Hall (recipient of a RF Fellowship) was appointed Senior Sanitary Medical Officer in the 1930s; the first Jamaican to hold a senior post in the Jamaican medical department (or indeed in any of the BWI).

25. James Riley, *Poverty and Life Expectancy. The Jamaican Paradox* (Cambridge: Cambridge University Press, 2005), 74; Gisela Eisner, *Jamaica, 1830–1930. A Study in Economic Growth* (Manchester: Manchester University Press, 1961), Table VI, 137.

26. Rockefeller Archives Center (RAC): Record Group (RG) 1.1 Projects, 437 Jamaica, Box 1 Folder 5, Report on Jamaica Programme for 1933, H.H. Howard, 5. As well as hookworm, the RF supported public health education, training, a yaws campaign, tuberculosis work and founded the first parochial health unit.

27. Note that malaria was first identified as a separate category of fever in 1882 but because of the lack of medical certification of deaths many if not most of the other fever deaths were most probably malarial.

28. Prout had worked in the West African medical service and was then a member of the Advisory Medical and Sanitary Committee at the Colonial Office. This committee had been set up in 1909 principally then to advise on medical matters in the African colonies. In 1922 its remit was extended to all colonies of the dependent Empire. TNA: PRO, CO 859/60/10, A History of the Colonial Advisory Medical Committee, R.B. Gray, 22 July 1941.

29. ProutW.T., ‘Reports of the Twenty First Expedition of the Liverpool School of Tropical Medicine, Jamaica 1908–9, Section II, Malaria’, Annals of Tropical Medicine and Parasitology, III, 4 (1909), 471–552, 486.

30. *Ibid.*, 492.

31. Prout, *op. cit.* (note 29), 495.

32. Prout, *op. cit.* (note 29), 493.

33. Prout, *op. cit.* (note 29), 498, 499.

34. Prout, *op. cit.* (note 29), Plate XVIII.

35. Snowden, *op. cit.* (note 13), chs 3 and 4.

36. Prout, *op. cit.* (note 29), 531. He thought it could be given to children in the form of ‘chocolate comfits’ as it was in Italy.

37. Prout, *op. cit.* (note 29), 534–5.

38. Prout, *op. cit.* (note 29), 536.

39. TNA: PRO, CO 140/241, Report of the Malaria Commission for year ending 31 March 1912, 375.

40. *Ibid.*, 377.

41. CO 140/241, *op. cit.* (note 39), 378.

42. TNA: PRO, CO 140/244, Report of the Island Medical Department 1913, 285.

43. TNA: PRO, CO 140/241, Report of the Malaria Commission, 379.

44. TNA: PRO, CO 140/237, Report of the Island Medical Department, 1910, 218.

45. This was certainly the reason given by the SMO in 1925; TNA: PRO, CO 140/265 Report of the Island Medical Department 1925, 269.

46. Jamaica National Archives (JNA), IB/5/77/282 Malaria Campaign 1926–7, Governor to Legislative Council, 25 September 1927.

47. ‘Arrival of the Malarial Expert’, *The Daily Gleaner*, 10 January 1928, 1. The delay was owing to the difficulty the RF had in obtaining the malariologist, JNA IB/5/77/282 Malaria Campaign 1926–7, Howard to Superintending Medical Officer, 2 June 1927.

48. BoydMark F. and ArisF.W., ‘A Malaria Survey of the Island of Jamaica, B.W.I’, American Journal of Tropical Medicine, 1–9, 5 (1929), 309–399: 392, 361, 364.

49. *Ibid.*, 392. A similar problem occurred with migratory labour in Italy. See Snowden, *op. cit.* (note 13), 25–6.

50. Boyd and Aris, *op. cit.* (note 48), 366–87. It had proved difficult to get someone to do this work. A Dr March who was appointed Boyd’s deputy in March 1928 resigned after two days. ‘He objected to wading in swamps in the manner prescribed by Dr Boyd. Dr Aris was then offered the post and as a trial was taken by Boyd to St Thomas “for the purpose of seeing ‘how he takes to the water””, RF RG 2 General Correspondence, 1928, 433, Box 10 Folder 81, B Washburn to H. Howard, 17 March 1928.

51. Boyd and Aris, *op. cit.* (note 48), 394.

52. Boyd and Aris, *op. cit.* (note 48), 355.

53. Boyd and Aris, *op. cit.* (note 48), 389–90.

54. Boyd and Aris, *op. cit.* (note 48), 391.

55. Boyd and Aris, *op. cit.* (note 48), 397.

56. Boyd and Aris, *op. cit.* (note 48), 397.

57. Boyd and Aris, *op. cit.* (note 48), 396–7; and see Snowden, *op. cit.* (note 13), 150–1.

58. Boyd and Aris, *op. cit.*, 397.

59. TNA: PRO, CO 140/272, Report of the Island Medical Department 1929, 284. Under Carley the SMO considered that the work at Annotto Bay had been initiated on a ‘firm basis’: TNA: PRO, CO 140/272, Report of the Island Medical Department 1929, 287.

60. TNA: PRO, CO 140/291, Report of the Malaria Officer, Report of the Island Medical Department, 1938, 278.

61. JonesM., Health Policy in Britain’s Model Colony (Hyderabad: Orient Longman, 2004), 203–208

62. Maternal and child welfare work, for example, was in the hands of voluntary providers who merely received a cash subsidy from the government.

63. World Health Organisation (WHO), M/2/372/3 Malaria Eradication Programme, Jamaica, Plan of Operations for malaria eradication program – Jamaica, 20 December 1957, 84; TNA: PRO, CO 137/880/7 CD and W, Drainage and Anti-Malarial Works, 1949, Minute, W. Bates, 18 November 1949.

64. TNA: PRO, CO 140/308, Report of the Island Medical Department, 1946, 3.

65. TNA: PRO, CO 140/316, Report of the Island Medical Department, 1949, 5.

66. TNA: PRO, CO 140/324, Report of the Island Medical Department, 1950, 8.

67. TNA: PRO, CO 140/335, Report of the Island Medical Department, 1951, 5. ‘Controlled areas’ were those deemed to be foci of malaria hence it would be expected that they would be higher even though anti-malaria measures were in operation.

68. TNA: PRO, CO 140/335, Report of the Island Medical Department, 1951, 5.

69. WHO7.077 Archives of the Parasitology Collection of the Communicable Diseases Documentation Centre at WHO Headquarters, 1911–2003, MALARIA-AMRO-JAMAICA, JKT 1, SJ3 1965, Register of Malaria Eradication of Jamaica, March 1965, 10; WHO7.077, Archives Parasitology, MALARIA-AMRO-JAMAICA, JK1 SJ1, 1950–8, United Nations Economic and Social Council, UNICEF, Recommendations of the Executive Director for an Allocation, Jamaica, 13 March 1957, 2. UNICEF gave $32,000 in aid in 1952.

70. MALARIA-AMRO-JAMAICA, JKT 1, SJ1 1950–1968, Public Health (Spraying) By-Laws, 1953; TNA: PRO, CO 140/337, Jamaica Report of the Medical Services, 1953, 3.

71. Third Generation Files, WHOHQ, M2/372/3 Jamaica, MEP, Dr H.M. Johnston to Dr C.A. Alvarado, Chief Malaria Division, WHO, Geneva, 10 August 1963, The Quartan Problem in Jamaica, 1.

72. Packard, *op. cit.* (note 3), 252–154; Cueto, *op. cit.* (note 15), 46–48.

73. Third Generation Files, WHOHQ, M2/372/3 Jam, Programme for Malaria Eradication in Jamaica, PASB/WHO Inter-Office Memorandum, Plan of Operations for malaria eradication program-Jamaica, 20 December 1957, 2–3.

74. WHO7.0091 MALARIA-AMRO-WEST INDIES 1936–82, JKT 1 SJ1 1936–55, Pan American Sanitary Bureau Regional Office of the WHO. Notes on Malaria Control in the Americas (1954) with estimates of the cost of Eradication, Washington DC, 24 March 1955, 2.

75. WHO7.077, Parasitology Archives, Recommendations of the Executive Director for an Allocation, Jamaica, 13 March 1957, 2–5.

76. Plan of Operations for malaria eradication programme – Jamaica, 20 December 1957, 106.

77. WHO7.077 Parasitology Archives, Malaria Eradication Training Centre Newsletter, January 1964.

78. Third Generation Files, WHOHQ, M2/372/3 JAM, Ross-Nicol, 17 March 1960.

79. Report for period 9 January–15 March 1960, 6. Someone at the WHO HQ had noted in the margin that telephone consultations would leave no written record. Bureaucracy demanded such paperwork presumably at the expense of the actual work.

80. Report, *op. cit.* (note 79), 2.

81. Report, *op. cit.* (note 79), 1.

82. Report, *op. cit.* (note 79), 3–4.

83. Third Generation Files, WHOHQ, M2/372/3 JAM, Ross Nicol, Report for period 16–31 March 1960, 1.

84. Third Generation Files, WHOHQ, M2/372/3 JAM, Bernard A. Ferri, Sanitarian, Annual Report Jamaica, 27 November 1961, 1.

85. Ross Nicol, Report for period 9 January–15 March 1960, 8.

86. Dieldrin had longer lasting residual effects but was more expensive than DDT. It is not clear why this more costly insecticide was chosen initially.

87. WHO7.077 Parasitology Archives, Register of Malaria Eradication, 13.

88. Cueto, *op. cit.* (note 15), 48.

89. Ross Nicol, Report for period 9th January–15th March 1960, 9.

90. *Ibid.*, 6.

91. Third Generation Files, WHOHQ, M2/372/3 JAM, Ross Nicol, Report for period 16–31 March 1960, 1.

92. *Ibid.*, 3.

93. Report, *op. cit.* (note 91), 4.

94. WHO7.077 Parasitology Archives, Register of Malaria Eradication, 16.

95. Third Generation Files, WHOHQ, M2/372/3 JAM(b), Meeting held on 21 March 1960, 1.

96. Ross Nicol, Report for period 16–31 March 1960, 3.

97. Third Generation Files, WHOHQ, M2/372/3 JAM(b), Meeting held on 21 March 1960, 2–3.

98. Third Generation Files, WHOHQ, M2/372/3 JAM, Programme for Malaria Eradication, Abraham Horwitz, Director PAHO, to Robert Davies, Regional Director, UNICEF, 26 September 1961, 2.

99. Third Generation Files, WHOHQ, M2/372/3 JAM(b), Ross Nicol, First Quarterly Report, January, February, March 1962, 8 May 1962, 1.

100. *Ibid.*, 1.

101. WHOHQ, M2/372/3, *op. cit.* (note 99), 3.

102. WHOHQ, M2/372/3, *op. cit.* (note 99), 3.

103. Third Generation Files, WHOHQ, M2/372/3 JAM(b), Ross Nicol, 1 May 1962, 2.

104. *Ibid.*, 2.

105. WHOHQ, M2/372/3, *op. cit.* (note 103), 2.

106. This was a point made to the Moyne Commission by Dr O’Brien, the CO medical adviser in 1938: NA: CO 950/47, Dr Arthur O’Brien, Memorandum on the Medical Services, Questions, 57.

107. Third Generation Files, WHOHQ, M2/372/3 JAM, Jamaica-2 Ross Nicol, Report for period 9 January–15 March 1960, 4–5.

108. *Ibid.*, 5, 6–7.

109. WHO7.077, Parasitology Archives, JK1 SJ2, 1958–1965, H.M. Archibald, Report of a Visit to Study Malaria Eradication in Mexico, Venezuela and Jamaica, 1959, 9.

110. See Sanjoy Bhattarcharya, *Expunging Variola. The Control and Eradication of Smallpox in India 1947–1977* (New Delhi: Orient Longman, 2006) for an account of the WHO smallpox eradication campaign in India for full exploration of these issues.

111. Register of Malaria Eradication of Jamaica, March 1965, 23.

112. Packard, *op. cit.* (note 3), 159.

113. Cueto, *op. cit.* (note 15), 164.

114. Boyd and Aris, *op. cit.* (note 48), 343–4.

115. Third Generation of Files, WHOHQ M/2/372/3 JAM, Jamaica II- Report for Period, 16–31 March 1960, 4.

116. This took the form of literature, demonstrations, film shows and so on.

117. Packard, *op. cit.* (note 3), 160.

118. Riley, *op. cit.* (note 25), 136.

